# Extraction of chitosan and its oligomers from shrimp shell waste, their characterization and antimicrobial effect

**DOI:** 10.14202/vetworld.2017.170-175

**Published:** 2017-02-12

**Authors:** Tarun Kumar Varun, Swaraj Senani, Natasha Jayapal, Jayaram Chikkerur, Sohini Roy, Vijay Bhasker Tekulapally, Mayank Gautam, Narender Kumar

**Affiliations:** 1 Department of Animal Nutrition, ICAR-National Dairy Research Institute, Karnal, Haryana, India; 2 Department of Animal Nutrition, ICAR-National Institute of Animal Nutrition and Physiology, Adugodi, Bengaluru, Karnataka, India; 3 ICAR-Indian Veterinary Research Institute, Izatnagar, Bareilly, Uttar Pradesh, India

**Keywords:** chitin, chitooligomers, chitosan, Fourier transform infrared, shrimp waste

## Abstract

**Aim:**

The present study was performed to utilize the shrimp shell waste for chitin and chitosan production, characterization by Fourier transform infrared (FT-IR) technique and to evaluate the antimicrobial effects of chitosan oligomers produced by depolymerization of chitosan by nitrous acid.

**Materials and Methods:**

Chitosan was extracted from the shrimp shell waste by the chemical method and characterized by FT-IR. Chitooligomers were produced by depolymerising chitosan using nitrous acid, and the chitooligomers were tested for antimicrobial effect against four gut pathogenic organisms, i.e., *Enterobacter aerogen* (National Collection of Dairy Culture [NCDC] 106), *Enterococcus faecalis* (NCDC 119), *Escherichia coli* (NCDC 134), and *Staphylococcus aureus* (NCDC 109) by well diffusion method using Muller-Hinton agar. A pure culture of pathogenic organisms was collected from NCDC, ICAR-National Dairy Research Institute, Karnal.

**Results:**

Extracted chitosan characterized by FT-IR and chitooligomers demonstrated antimicrobial effect against four gut pathogenic organisms used in this study. Zone of inhibitions (mm) were observed in *E. faecalis* (13±0.20), *E. coli* (11.5±0.4), *S. aureus* (10.7±0.2), and *E. aerogen* (10.7±0.3). *E. faecalis* showed larger inhibition zone as compared to all other organisms and inhibitions zones of *E. aerogen* and *S. aureus* were comparable to each other.

**Conclusion:**

Shrimp waste can be utilized for chitosan production, and the chitooligomers can be used as feed additive for gut health enhancement and have potential to replace antibiotics from the feed. Along with value addition pollutant load could be reduced by waste utilization.

## Introduction

India is the third largest producer of the fishery in the world and hence also produces a huge amount of fish waste. Shrimp production of India was 2.7 lakh MT (Table-1). Major groups in crustaceans landings were non-penaeid shrimps (67.50%) followed by penaeid shrimps (24.35%) [1]. Chitin is the second most abundant biopolymer after cellulose in nature. Estimated annual production of chitin by living organisms is 10^10^-10^12^ tons/year [2]. Structurally, chitin has acetamide group (-NHCOCH_3_) at C-2 position, and rest is similar to cellulose. Chitin is a natural polymer having monomeric unit of N-acetylglucosamine linked by β,1-4 glycosidic linkages. Chitosan is the deacetylated product of chitin and is linear, polycationic and heteropolysaccharide mainly composed of β-1,4-2-deoxy-2-amino-D-glucopyranose and β-1,4-2-deoxy-2-acetamido-D-glucopyranose glycosidic linkages [3]. If the content of N-acetyl-D-glucosamine in polymer is higher than the biopolymer is chitin and if the content of glucosamine is higher than the biopolymer is chitosan [4]. Major reactive functional groups are amino/acetamide group and primary and secondary hydroxyl group at C-2, C-3, and C-6 positions, respectively. Due to the amino group content of chitosan structural, physicochemical and biological properties differs [5]. Degree of deacetylation of chitosan widely varies between 75% and 95% and molecular weight between 50 and 2000 kDa [6]. Annual production potential of chitin in India from shrimp is 3560 tonnes [7]. Chitosan and its oligomers are known for its various biological properties such as antioxidant [8], anti-inflammatory [9], cholesterol lowering [10], immunity enhancing [11], antitumor [12], neuroprotective [13], antimicrobial [14], and antifungal [15] which makes chitosan and its oligomers very useful polysaccharide for animal health. Chitosan is insoluble in water, basic pH solutions and organic solvents which is a major limitation of chitosan. Chitosan is soluble in dilute organic acid like acetic acid, formic acid, etc., and form high viscous solution. Chitosan solubility depends on distribution of N-acetyl and free amino groups. Protonation of amino groups occurs at pH<6.0 due to which chitosan become soluble [16] but at basic pH protonation does not occur and pH value of ~6.5 leads to solubility and insolubility transition [17]. Recent studies revealed the conversion of chitosan to oligomers as oligomers are soluble in water. Chitooligomers are the depolymerized product of chemical and enzymatic hydrolysis. Depolymerization of chitosan can be carried out by nitrous acid deamination [15], flurolysis using anhydrous hydrogen fluoride [18], and oxidative reduction reaction using hydrogen peroxide [19]. Chitosan with molecular weight <39 kDa and degree of polymerization <20 are known as chitosan oligomers or chitooligomers [20]. In depolymerization of chitosan with nitrous acid (NaNO_2_+CH_3_COOH), the amino group of chitosan selectively reacts with nitrosyl cation and forms a diazonium salt which after releasing nitrogen becomes unstable carbocation which further rearranges itself and leads to cleavage of β,1-4 glycosidic linkage forming 2,5-anhydro–D-mannose and a detached polymer. This diazotization reaction continues until the sodium nitrite is completely consumed [15]. Mechanism of inhibition of microbial cells is by chitosan and its oligomers via its polycationic nature which electrostatically binds with the microbial surface and interferes with metabolism of bacteria or by blocking the transcription of RNA from DNA by adsorption on DNA after penetration to the cell. For penetration, the molecular weight of chitosan must be <5000 kDa [21]. Antimicrobial property of chitosan mainly depends on molecular weight and degree of deacetylation.

**Table-1:** State wise details of shrimp production.

State	2011-12	2012-13
	
	Estimated production (MT)	Estimated production (MT)
West Bengal	45,999	52,581
Odisha	11,001	14,532
Andhra Pradesh	1,26,466	1,59,083
Tamil Nadu	14,960	25,815
Kerala	8138	5175
Karnataka	841	664
Goa	51	63
Maharashtra	2662	3513
Gujarat	6065	9393
Total	2,16,183	2,70,819

Source: Handbook of fisheries statistics. 2014. DAHDF, Ministry of Agriculture, Government of India, p 69

In this study, shrimp shell waste was utilized for extraction of chitosan and its oligomers and effect of chitosan oligomers was observed on different pathogenic organisms.

## Materials and Methods

### Ethical approval

The experiment was conducted in accordance with the guidelines laid down by the Institutional Animal Ethics Committee for Animal Care and Management.

### Study area

The study was conducted in Animal Nutrition Division, Feed additives and Nutraceutical Lab at ICAR-National Institute of Animal Nutrition and Physiology, Adugodi, Bengaluru (Karnataka) in collaboration with ICAR-National Dairy Research Institute (NDRI), Karnal (Haryana).

### Bacterial strains

All pure bacterial strains used were taken from National Collection of Dairy Culture (NCDC), NDRI, Karnal (Haryana). Pathogenic bacterial strains used were *Escherichia coli* (NCDC-134), *Staphylococcus aureus* (NCDC-109), *Enterococcus faecalis* (NCDC-119) and *Enterobacter aerogen* (NCDC-106). All these bacterial strains were present in the lyophilized form in ampoules.

### Shrimp shell waste

Shrimp shell waste was procured from Russell fish market, Shantinagar, Bengaluru (Karnataka). Sample was taken fresh and taken to the laboratory within 1 h of procurement.

### Extraction of chitosan and oligomers

Shrimp shell waste was collected from the local fish market. Samples were taken and washed properly with flowing tap water to remove the soil and extraneous matter. Thoroughly cleaned sample was kept for drying at 80°C in hot air oven for 2-3 days. Dried sample was finely ground in a grinding machine and kept in the air tight container. Compositional analysis of sample was performed as per AOAC [22]. Minerals were analyzed by Inductively Coupled Plasma Opti­cal Emission Spectrum (ICP-OES, Optima 8000 M/s Perkin Elmer, USA).

### Extraction of chitin and chitosan from shrimp shell waste

For extraction of chitin, the conventional chemical method was followed. Chitosan extraction was done following three major steps, i.e., demineralization, deproteination, and deacetylation. For demineralization, 10 g of sample was treated with 2N hydrochloric acid at solid to solvent ratio of 1:15 for 2 h with constant stirring at 150 rpm in incubator shaker at room temperature [23]. Acid was slowly added to avoid frothing due to gas formation occurring because of calcium carbonate content of shell which reacts with the acid and form carbon dioxide. After demineralization, the sample was washed with tap water till the sample reaches neutral pH. Final wash was given with hot distilled water and sample was kept for drying at 80°C overnight.

**Figure-1:**
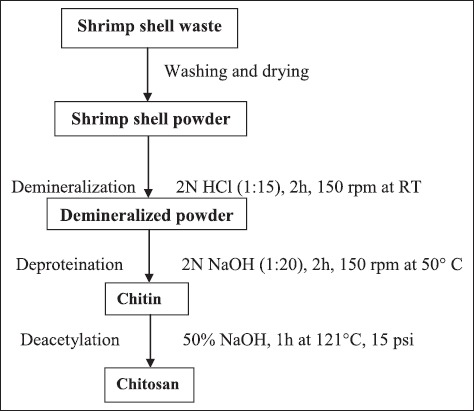
Flow diagram showing the steps for extraction of chitin and chitosan from shrimp shell waste.

For deproteination, demineralised shrimp shell powder was treated with 2N NaOH at solid to solvent ratio 1:20 for 2 h with constant stirring at 150 rpm at 50°C in an incubator shaker [23] followed by thorough washing and drying as mentioned above. After this step, the end product was chitin. For deacetylation, chitin was treated with strong alkali, i.e., 1 g of chitin was added to 50% NaOH for 1 h at 121°C, 15 psi followed by washing till it reaches neutral pH. After drying, the final product recovered was chitosan (Figure-1).

### Characterization of extracted chitin and chitosan

Extracted chitosan was confirmed by solubility test in dilute acetic acid and characterization was done using Fourier transform infrared spectroscopy (FT-IR) in which spectra of standard chitosan and extracted chitosan were obtained and on the basis of absorption spectra of the standard chitosan the sample were analyzed and characterized.

### Chitosan oligomers production

Hydrolysis of chitosan was done by nitrous acid (NaNO_2_+CH_3_COOH) for oligomers production. For hydrolysis, 2% chitosan solution in dilute 1% acetic acid and 0.5 M NaNO_2_ were added in 9:1 proportion and kept at room temperature in incubator shaker for overnight. pH was adjusted up to 6.0 using 10N NaOH. Depolymerization could be predicted by decrease in the viscosity of chitosan.

### Antimicrobial properties of chitosan oligomers

This property of chitosan oligomers was evaluated by well diffusion method. For the antimicrobial property, Muller-Hinton (M-H) agar was used. M-H agar was used at 38 g/L of media. This agar was divided into flasks for autoclave as per number of bacteria to be tested. After autoclave, agar was allowed to cool up to 40°C and then 1% of the cultured broth from mother culture was seeded into it and after mixing properly, it was poured into sterile Petri plates. After solidification of the agar, wells were punched by sterile borer aseptically under the laminar flow. 200 µl of neutralized hydrolysate containing oligomers filtered through 0.20 µm polyvinyl difluoride syringe filter was poured into the well. The plate was then kept in the refrigerator at 4°C for 1 h as this temperature is bacteriostatic thus during this time the oligomers can diffuse into the agar. Afterward, plates were kept at 37°C for 24 h and the diameter of zone of inhibition was measured. Blank was also prepared in the same way without chitosan. This is a preliminary study, and positive and negative control will be included in the future study. The test was done in triplicates.

## Results

The shrimp shell waste contained 27.53±0.71 (%) dry matter (DM). Total ash, crude protein, ether extract and crude fiber (CF) (% DM basis) were 20.02±0.09, 50.83±0.018, 4.51±0.09 and 12.03±0.35 (%). CF values showed close proximity with the amount of chitin and chitosan produced from shrimp shell waste sample. Mineral composition reveals that the amount of macro minerals, i.e., calcium, phosphorous, and magnesium (%) were 9.08±0.32, 1.37±0.05 and 0.46±0.05 and the amount of micro minerals, i.e., copper, zinc, iron and manganese (ppm) were 105.44±42.28, 58.91±1.26, 201.47±11.64 and 17.91±0.68, respectively. Yield (%) of chitin and chitosan from the shrimp shell waste was 14.72±0.57 and 12.03±0.46, respectively. Formed chitosan was almost soluble in the 1% acetic acid and was characterized using FT-IR technique and compared with the wavelength bands of the standard chitosan.

**Figure-2:**
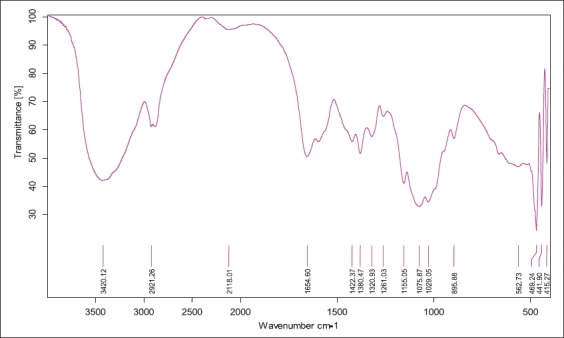
Fourier transform infrared of standard chitosan.

**Figure-3:**
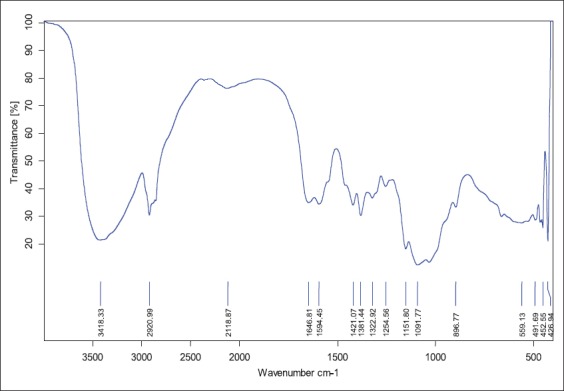
Fourier transform infrared of shrimp chitosan (extracted).

FT-IR spectrum of extracted chitosan from shrimp showed peaks at 3418 cm^-1^ that indicated stretching vibration of - hydroxyl group, -NH_2_ group of amines and hydrogen bonding which was comparable to spectrum peak of standard, i.e., 3420 cm^-1^. 1646 cm^-1^ peak in extracted chitosan indicated the vibrations of carbonyl group (amide band I) and standard had this peak at 1654 cm^-1^. Peaks at 1594 cm^-1^ (extracted chitosan) and 1580 cm^-1^ (standard) showed the presence of amide band II (N-H bendings). For -CH_2_ groups’ in CH_2_OH, peaks were observed at 2921 and 1422 cm^-1^ in standard which overlapped with band spectrum at 2920 and 1421 cm^-1^ in shrimp chitosan. -CH_3_ group of NHCOCH_3_ (amide bond) was shown at 1380 cm^-1^ in standard and at 1381 in shrimp chitosan. Oxygen stretching of glycosidic linkage was found at 1155 cm^-1^ in standard but in shrimp chitosan it was found at 1151 cm^-1^. Pyranose ring was found at 895 cm^-1^ in standard and in shrimp chitosan it was at 896 cm^-1^. As the deacetylation process occurred, there was a variation in the intensity of carbonyl group at 1655 cm^-1^ and amide band peak at 3449 cm^-1^. Glycosidic linkage indicated by peak at 1151 cm^-1^ in shrimp chitosan which overlapped with standard chitosan at 1155 cm^-1^. The presence of CH_3_, CH_2_ and CH groups as well as the primary and secondary-OH groups which are attached to the pyranose ring, represented by the spectra between 1422 and 603 cm^-1^ (Table-2, Figures-2 and 3). The presence of the entire band stretching in the extracted chitosan compared with standard band stretching depicts that extracted material was chitosan. Zones of inhibition test were employed to study the antimicrobial effect of chitosan oligomers. Zones of inhibitions observed for different bacterial strains under the study are shown in Table-3. This *in vitro* study presented the antimicrobial action of chitosan oligomers against four gut pathogenic organisms, i.e., *S. aureus*, *E. coli*, *E. faecalis*, and *E. aerogen*. As per reviewed literature, the antimicrobial effect is shown only if molecular weight of chitosan oligomers is <5000 kDa, therefore, the depolymerized chitosan formed in this study should be <5000 kDa.

**Table-2:** Wavelength of the bands obtained by the FT-IR of extracted chitosan from Shrimp.

Vibration mode indicating various bonds in the compound	Std. chitosan (cm^−1^)	Extracted shrimp chitosan (cm^−1^)
(NH_2_) assoc. in primary amines (OH) assoc. in pyranose ring	3420	3418
(CH_2_) in CH_2_OH group	2921	2920
(C=O) in NHCOCH_3_ group (Amide I band)	1654	1646
Amide II band (N-H bending)	1580	1594
(CH_2_) in CH_2_OH group	1422	1421
(CH_3_) in NHCOCH_3_ group	1380	1381
Amide III band (C-N stretching)	1320	1322
Asymmetric bridge oxygen stretching (glycosidic linkage)	1155	1151
(C-O) in secondary OH group	1075	1071
(C-O) in primary OH group	1029	1020
Pyranose ring stretching	895	896

FT-IR=Fourier transform infrared

**Table-3:** Inhibitory effect of chitooligomers against pathogenic organism.

S. No.	Bacteria	Zones of inhibitions (mm)
1	*Enterobacter aerogen* (NCDC 106)	10.7±0.3^b^
2	*Enterococcus faecalis* (NCDC 119)	13±0.20^a^
3	*Escherichia coli* (NCDC 134)	11.5±0.4^ab^
4	*Staphylococcus aureus* (NCDC 109)	10.7±0.2^b^
	p value	<0.05

Each mean value is average of four replicates. ^a, b^Means bearing superscript differ significantly. NCDC=National Collection of Dairy Culture

## Discussion

This study aimed at the utilization of shrimp shell waste for production of chitosan and its oligomers. Abdulkarim *et al*. [24] reported 15% yield of chitosan from shrimp shell waste which is slightly higher than yield of chitosan in study, i.e., 12.03%. Recovery of chitosan in the present study from shrimp waste is 12.03% was far superior as compared to chitosan yield from shrimp shell waste reported by Isa *et al*. [25], i.e., 8.15%. The variation could be due to difference in the age of the shrimps from which the sample was taken. FT-IR analysis of chitin and chitosan showed the band spectrums of different bonds in the present study were in close agreement with the Brugnerotto *et al*. [26] who observed bonds at 3436 cm^-1^ of hydroxyl group, 1661 and 1315 cm^-1^ for amide I and amide II bonds, respectively. Zakaria *et al*. [27] also reported similar results; the band spectrum for hydroxyl group and –NH_2_ at 3438 cm^-1^ and amide bands between 1639 and 1561 cm^-1^ which were in close agreement with band spectrums obtained in this study.

Chitooligomers have been reported to inhibit many bacteria. Antimicrobial activity of chitosan oligomers may be due to interference in the metabolism by binding to the surface of the bacteria or by blocking of transcription of DNA and RNA by binding to the DNA after penetration into cell. Chitooligomers showed antimicrobial action against pathogenic organisms and significantly inhibited *E. faecalis* and the inhibitory effect on *E. aerogen* and *S. aureus* was comparable. Chitooligomers also inhibited *E. coli* which is one of the main gut pathogenic organisms. Benhabiles *et al*. [23] showed antimicrobial effect of chitooligomers against many pathogenic organism, i.e., *S. aureus, Salmonella typhimurium, Bacillus subtilis, Bacillus cereus*, and *Vibrio cholera* at 0.1% concentration of chitooligomers and chitosan. Similarly, Fernandes *et al*. [28] showed the antibacterial effects of chitosan and chitooligomers against *S. aureus* and *E. coli* at 0.1%, 0.25% and 0.5% concentrations. Simunek *et al*. [29] showed the antimicrobial effect of chitooligomers on different species of *Bifidobacterium*. *Bifidobacterium adolescentis*, and *Bifidobacterium longum* were inhibited by the chitooligomers at concentration from 0.025% to 0.5%. Shanmugama *et al*. [30] reported similar results about the antibacterial effect of chitosan on Gram-positive bacteria *S. aureus* and Gram-negative bacteria *E. coli* at various concentration of chitosan.

## Conclusion

This study revealed that shrimp shell waste could be effectively utilized for the extraction of chitin, chitosan, and chitooligomers. *In vitro* study exhibited antimicrobial action of chitooligomers against many gut pathogens and hence could enhance the gut health. Thus, chitooligomers as feed additive may replace antibiotics in the animal feed which in turn help in dealing with problems of antibiotic residue in the animal products.

## Authors’ Contributions

TKV: Carried out all experimental and laboratory work. SS: Planned, guided and supervised the entire research work. NK: Helped in sample collection and its proximate analysis. NJ, SR, JC, MG helped in lab experiments and preparation of the manuscript. TVB: Carried out the data analysis. All authors have read and approved the final version of manuscript.
